# An evaluation of North Carolina science advice on COVID-19 pandemic response

**DOI:** 10.1057/s41599-022-01344-9

**Published:** 2022-10-05

**Authors:** Jessica Weinkle

**Affiliations:** University of North Carolina Wilmington, Wilmington, NC USA

**Keywords:** Politics and international relations, Science, technology and society

## Abstract

This qualitative case study contributes to the international research project EScAPE (Evaluating Scientific Advice in a Pandemic Emergency) and aims to understand how state leaders mobilized science advice in pandemic response during 2020 and into the early months of 2021. North Carolina, a state in the southeastern United States, mobilized much of its pandemic science advice through the state’s Department of Health and Human Services. A fluid relationship between advisors and the governor—credited as a crucial component of a science driven, balanced pandemic response—created an opaque hub of advising and power. I analyze three advisory processes apparent during early stages of pandemic response noting strengths in mutual respect and trust between advisors and policymakers, data transparency, and commitment to equitable vaccine distribution. The interpersonal dynamics that provided these “good” science advice outcomes are a result of the individuals involved but the dynamic is not guaranteed in government over time. Also, while North Carolina provided data transparency it is unclear how data trends connected to decisions. There is a general lack of transparency around the breadth and content of advice. Transparency of advisory mechanisms is important to maintain public trust in government. Deep partisanship in the United States and distrust between leaders of opposing parties underscores the need for states to develop strong institutions for science advise to policymakers in an emergency. This article closes with several recommendations.

## Introduction

North Carolina state government developed plans for influenza pandemic response well before the spread of COVID-19. These plans did not include a science advisory mechanism likely due to central assumptions within the plans about the coherence and competence of international and federal agencies to provide requisite guidance on the state of knowledge. During 2020, scientific knowledge about COVID-19 was in flux and assimilated within the North Carolina Department of Health and Human Services (NCDHHS) from a wide range of formal and informal sources.

North Carolina developed influenza pandemic plans around 2006 under the guidance of the George W. Bush Administration in response to the H5N1 influenza (bird flu) pandemic threat (NCDHHS, 2019; US Homeland Security Council, 2005). A major update to the plan occurred in 2009 in response to the H1N1 influenza (swine flu) pandemic. Another update was underway when it was derailed by the COVID-19 pandemic response in 2020 (NCDHHS, personal communication, October 23, 2021). Overall, the North Carolina influenza pandemic plans outline a chain of command, processes of resource and information distribution, and provide clarification on the legal authority of the state to restrict the public’s civil liberties (NCDHHS, 2019). The state’s chain of command begins at the global level by recognizing the authority of the World Health Organization (WHO) to coordinate international disease surveillance. Then, it recognizes the US Government as the next entity in line for coordinating response.

North Carolina’s plans make a key assumption about information coherence, “The World Health Organization (WHO) and CDC [US Center for Disease Control and Prevention] will coordinate surveillance at the national and international level” (NCDHHS, 2019, Part B, p. 2). The state would then be responsible for providing guidance to counties and localities,

Counties and local health departments will rely on state guidance, leadership and resources to continue critical functions. In turn, the state will rely on guidance, leadership and resources from the federal government.

State plans emphasized leadership of the US Centers for Disease Control and Prevention (CDC) to augment state resources with necessary expertize in: (1) disease surveillance, (2) epidemiological response, (3) diagnostic laboratory services and reagents, (4) education and communication, and (5) disease containment and control (NCDHHS, 2019, Plan A, p. 1).

Included in North Carolina’s pandemic plans is an “Ethics Report” providing ethical guidelines for response to an influenza pandemic including the allocation of scarce resources such as vaccines (NCIOM, 2007). A central message of the Ethics Report is the importance of clarity and transparency around information as a foundation for building and maintaining public trust in government prior to and during a pandemic,

Government should disseminate information via the media and trusted community leaders to help ensure that information reaches people at risk. Providing timely and accurate information will help reduce the spread of misinformation and panic (NCIOM, 2007, p. 15).

The Ethics Reports is clear that information alone is not sufficient for managing the social conflicts likely to arise from trade-offs between public health and civil liberties. Rather, decision makers would also need to provide clear reasoning and enlist others with grassroots leadership roles,

Decision makers will be confronted with the challenge of maintaining the public’s trust while simultaneously implementing various control measures during an evolving health crisis. Trust is indispensable for expectations of compliance. Trust is enhanced by transparency in decision making, equity in the application of restrictions and/or allocation of limited resources and reciprocity toward those with an increased burden (NCIOM, 2007, p. 65)

Though the Ethics Report does not provide guidance on how to handle uncertainty arising from the scientific or political context it indicates that such uncertainty is likely and encourages the development of conflict resolution processes,

There should be opportunities to revisit and revise decisions as new information emerges throughout an influenza pandemic. There should be mechanisms to address disputes and complaints; however, the extent of the review process must be balanced with the need to make quick decisions in the midst of an influenza pandemic (NCIOM, 2007, p. 66)

It is not unreasonable that the state of North Carolina built their pandemic response plan on the expectation that the WHO, CDC, and federal government could and would provide coordinated and competent guidance on the state of knowledge about the public health threat. The assumption was encouraged by the Bush Administration and supported by the Barack Obama Administration (Diamond and Toosi, 2020). However, dysfunction within these centralilzed institutions meant that individual American states had to develop their own science advisory process for COVID-19 pandemic response.

This article presents a qualitative evaluation of processes of science advice to policymakers on pandemic response in North Carolina. I give credit to the role of the social and political context in shaping the opportunities and limits of science advice to influence policymaking (Pielke, 2007; Sarewitz, 2011). This research contributes to the work of an international collaboration of scholars assessing the performance of state and national mechanisms of science advice in the context of COVID-19 pandemic global crisis, Evaluation of Science Advice in a Pandemic Emergency (EScAPE, escapecovid19.org). Collectively, the 22 national, subnational, multilateral institution case studies provide a comparative, international evaluation of mechanisms of science advice.

This article proceeds as follows: First, I provide a basic introduction to United States federalism because political culture is important for understanding the development and use of science advice. This section also includes a brief legislative description of relevant roles and authorities held by the state governor and NCDHHS secretary. Second, I illustrate some of the actions of former President Trump in undermining the ability of federal science advisory processes to act authoritatively though I do not evaluate the soundness of those processes. Third, I use the scholarly literature to develop criteria of “good” science advice by which to judge the success of science advisory mechanisms in North Carolina. Fourth, I describe data, methods, and three avenues of science advice to North Carolina policymakers. Fifth, I make judgments of strengths and weaknesses of the three science advisory processes in relation to criteria of good advice and consider the overall picture of NCDHHS advising to the governor. Sixth and finally, I close with recommendations for improving science advisory processes in North Carolina for pandemic response.

## American federalism and partisan polarization: the political context of state pandemic response

The way nations approached science and expertize in COVID-19 pandemic response varied dramatically around the world reflecting the unique social and political context of each place (Christensen and Lægreid, 2022a; Bennouna et al., 2021). This section provides a brief overview of United States federalism and its interaction with deepening partisan polarization. Though federalism does not necessarily create instability in science advice the temperament of federalism at a given time plays a role in the opportunities for science advice to support policymaking coherence (Easton et al., 2022).

Negotiating the limits and arrangement of federal and state authority is a central political activity in the United States. The Constitution explicitly articulates the limited powers of the national^1^ government in the Tenth Amendment, “The powers not delegated to the United States by the Constitution, nor prohibited by it to the States, are reserved to the States respectively, or to the people.” The decision making dynamic resulting from this system of distributed power—federalism—is an important component of United States political culture and the states exhibit unique political subcultures reflecting their individual histories and economies (Elazar, 1990).

Since the 1970’s, elite polarization has driven partisan sorting among the electorate (Levendusky, 2009). Where parties once had a mix a mix of liberals and conservatives they are now uniform with liberal leaning voters concentrated in the Democratic party and conservative leaning voters in the Republican party. In turn, voters are more partisan and the parties are more ideological.

The current state of polarization creates challenges for policymaking through federalism (Konisky and Nolette, 2021). Members of opposing parties distrust one another and distrust government when the opposing party is in power (Abramowitz and Webster, 2016). Distrust undermines the abilities of government to develop effective policies that address public needs (Hetherington and Rudolph, 2015). State governors are partisan in their decision making showing loyalty to national level party leaders over their state electorate (Jensen, 2017). The result is an acrimonious “uncooperative federalism” whereby states with governors loyal to one party actively resist and challenge federal policy implemented under presidential leadership of the opposing party (Bulman-Pozen and Gerken, 2009).

### Federalism, party polarization, and pandemic science advice to the US President

United States public health policymaking grapples with a tension between collective distaste for public control over private behavior and the desire to limit detrimental effects on the public from private actions (Oliver, 2006). Polarization, federalism, and the divisive 2020 general election year is the political backdrop on which trade-offs in public health policymaking were negotiated for pandemic response. The national government has authority to control movement across the country’s boarder and order individuals entering the country to isolate or quarantine, but it is an exclusive state power to issue population level stay at home orders and business closures (CDC, 2020). Thus, creating a national “shut down” required state governments to oblige federal guidelines on limiting public movement.

The incumbent Republican President Donald Trump, a billionaire and reality television star, rose to popularity in 2016 upon a populist ideology that equates expertize to elitism and devalues positivistic claims (Head and Banerjee, 2020). During his term President Trump interfered with official science advisory processes and undermined the legitimacy of several national institutions (e.g., “Sharpiegate,” Sobczyk, 2020). His attack on institutional legitimacy culminated in his efforts to erode public trust in the election process declaring before, during, and after the 2020 general election that the only way he could lose was if the election was “rigged” (quoted in Chalfant, 2020). After losing the election, a mob attacked the Capital Building presumably to halt the Congress’ confirmation of Democrat Joe Biden’s election win. There is much discussion about the potential role President Trump had in leading, or failing to discourage, the attack (H. Resolution 503, 2021).

As the public became more aware of the spread of COVID-19 in early 2020, the president downplayed the threat publicly announcing in February that, “Looks like by April, you know, in theory, when it gets a little warmer, it miraculously goes away” (quoted in Wise, 2020).^2^ By March, the federal government issued an advisory encouraging people to stay at home and avoid gatherings for 15 days, which was then extended to 45 days.

Eventually, all states chose to implement mandatory stay at home orders along federal guidelines. By the end of April the president noted that federal social distancing guidelines were “fading out, because now the governors are doing it” (quoted in Ordoñez, 2020). Mandatory orders continued to be issued by states on a variable basis over the course of 2020 and well into 2021. As state’s chose their own paths, President Trump likened governors’ stay at home orders to oppressive regimes,

“LIBERATE” Minnesota, Michigan and Virginia—all states where aggrieved residents have gathered in public in recent days to demonstrate in opposition to stay-at-home orders declared by Democratic governors” (quoted in Forgey, 2020a).

Tension grew between the president and public health authorities becoming palpable by April. During an interview on public television, Dr. Anthony Fauci, director of the National Institute of Allergy and Infectious Diseases, emphasized the importance of wearing a face mask to limit the spread of the disease. The next day, President Trump simultaneously announced and dismissed CDC recommendations to wear face masks, “I won’t be doing it, personally. It’s a recommendation” (CBS News, 2020). By June, the face mask had become a political symbol: Democrats sang its praises and Republicans publicly rebuffed its merits (Hellmann, 2020).

**Fig. 1:**
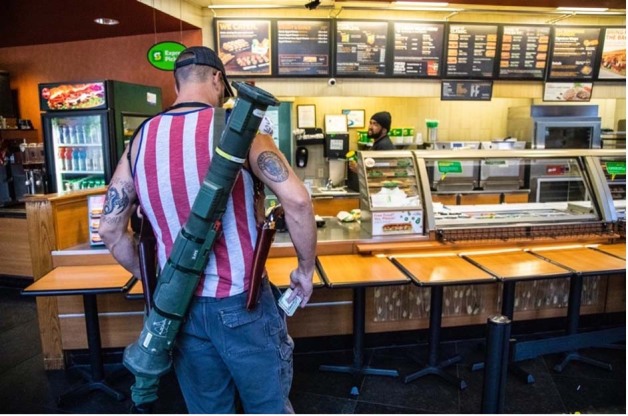
Armed protester in a restaurant. Boogaloo Boys protester wearing a rocket launcher and two pistols. Photo by Travis Long. This figure is not covered by the Creative Commons Attribution 4.0 International License. Reproduced with permission of Raleigh News & Observer; copyright © Raleigh News & Observer, all rights reserved.

In North Carolina, Republican legislators challenged executive orders limiting social gathering that were issued by the state’s Governor Roy Cooper, a Democrat. The conflict between the governor and General Assembly about appropriate pandemic response reflected national level party polarization and conflict transpiring on the ground. Grassroots organizations considered social control mandates as unduly infringing on civil liberties. Some argued that their purpose is to defend the Constitution with violence, if necessary, “Are we willing to kill people? Are we willing to lay our lives down? We have to say yes” (quoted in Wiseman, 2020). Boogaloo Boys, a far-right extremist group seeking an insurrection against the national government (The Economist, 2020), took to the streets adorned in military style weapons and clothing to protest the governor’s social distancing orders (Fig. 1, Banov and Long, 2020).

Public trust in the CDC plummeted as reports surfaced of the Trump Administration intervening with data collection, analysis, and advisory processes (Florko, 2020). President Trump publicly attacked CDC recommendations,

I disagree with the @CDCgov on their very tough & expensive guidelines for opening schools. While they want them open, they are asking schools to do very impractical things. I will be meeting with them!!! (quoted in Sprunt and Turner, 2020).

Though not providing the menacing imagery of extremist groups, teachers associations were also forceful. When Governor Cooper permitted school districts to reopen elementary schools the North Carolina Association of Educators (NCAE) claimed to be “forced to fight” for more stringent classroom social distancing measures and threatened a strike (Hui and Innis, 2020). As middle and high schools were set to reopen NCAE challenged CDC reopening guidelines arguing that it had not demonstrated that such changes were “justified by the science” and that a deeper explanation was needed “for the sake of public trust and clarity” (Hui, 2021).

By October, President Trump’s animosity towards federal science advisors was well established and reports surfaced that he regarded all of them poorly,

People are tired of Covid. People are saying, ‘Whatever, just leave us alone.’ People are tired of hearing Fauci and all these idiots (quoted in Forgey, 2020b).

It became unclear what science advising to the president was taking place, if any. When the new Democratic administration took office, Dr. Deborah Birx, pandemic response coordinator for the Trump Administration Coronavirus Task Force, revealed the depths to the lack of transparency on where and how Trump received expertize about the pandemic as she herself had no idea (Hooper, 2021).

## What is good science advice?

Public trust in government is in decline around the world (OECD, 2017). Most public issues have a significant technical component involving mechanisms of science advice. Public trust in government suffers when science advice lose integrity whether due to research misconduct, scientists’ overreach in politics, or policymaker meddling in research reporting (e.g. Jasanoff, 1998; Phillips et al., 2000; Chan and Ridley, 2021). Mistrust undermines implementation of policies and programs intended to improve public welfare. Goldenberg (2021) documents how histories of research misconduct and discrimination has led to vaccine hesitancy among a non-negligible portion of the population.

Public and policymaker trust in official science advisory mechanisms is an important resource for facilitating coordinated and sustained crisis response (Cairney and Wellstead, 2021). Crisis response is often accompanied by a centralization of power and greater “blurring” in the division between advisors and political decision making (Christensen and Lægreid, 2022b). Transparency in advice and the values underpinning policy decisions are important for bolstering public acceptance of the blurred boundary.

Transparency in science advice—both in its process and epistemic substance—contributes to democratic accountability by making the knowledge base for decisions apparent and ensuring decision making responsibility remains with those having the consent of the governed. Towards ensuring a transparent relationship between science advisors and policymakers, scholars and practitioners of science advice identify principles of “good” advice (e.g., Lentsch and Weingart, 2011, identify four principles and Gluckman, 2014, identifies ten principles). Common among these are two basic principles that relate science advising to democratic policymaking. First, science advisors must not lose sight that their work is part of the broader political process of policymaking and therefore, advice must remain neutral in regards to policy preference. Neutrality protects advisors and the disciplines they represent from appearing to circumvent democratic process and the public will. Moreover, neutrality improves the ability of the public to hold policymakers accountable for decisions by making clear that policymakers have choices. Second, and related to the first, good science advice fosters trust among those in government, media, and the public. Science advice to policymakers preserves public trust in the policy process when it provides transparent, independent, and sound assessment of scientific information, broadens the scope of policy options, and places responsibility for decision making with designated policymakers (Pielke, 2007). By some accounts good science advice is not about giving “advice” in the conventional sense of telling someone what they ought to do. Rather, good science advice provides a synthesized account of the state of knowledge, the values that underpin scientific evidence, and a range of options for action to empower policymakers to act and empower the public to hold policymakers accountable for their actions (Doubleday and Wilsdon, 2013).

A common conception holds a clean division between the activities of science advice and politics but in practice “the intermixing of politics and science is endemic” (Pielke and Klien, 2010, p. 156). As a novel disease, scientific knowledge about COVID-19 was uncertain. The social problem of pandemic response was highly complex, requiring trade-offs among social values of civil liberties, public health, education, and community. In such contexts it is difficult if not impossible to insulate the development of scientific knowledge from politics. The research questions asked and the methodologies employed correspond to value prioritizations and beliefs (Funtowicz and Ravets, 1993). The higher the degree of scientific uncertainty and value conflict the more important it is for science advisors to present the state of knowledge so that it creates decision options and makes apparent the value prioritizations represented by those options (Pielke, 2007). Doing otherwise risks invoking “inappropriate expertize” whereby scientists mask the political content of their claims by “appearing to be speaking the language of science” (Sarewitz and Rayner, 2021).

Pielke (2007) presents four idealized roles of scientists in society that provides a means for understanding processes of science advice and their outcomes, and the choices scientists face when providing advice to policymakers. These four roles are as follows:

*Pure Scientist* focuses on research with no consideration for its utility

*Issue Advocate* focuses on implications of research (i.e., methods, data, results) for a particular political agenda and directly aligns themselves, though perhaps implicitly, with an interest group

*Science Arbiter* responds to decision makers’ positive questions and avoids normative questions

*Honest Broker* engages in decision making by clarifying and perhaps expanding the options available to decision makers

Advisors should be honest with themselves and others about the role they are playing when offering advice. When science advisors claim the role of Science Arbiter or Honest Broker but play the role of Issue Advocate it displaces power for decision making, makes it difficult to hold policymakers accountable for decision outcomes, and undermines public trust in the policymaking process.

## Orienting inquiry and methods

This analysis focuses on mechanisms of science advice to policymakers specifically, Governor Roy Cooper. The governor is a natural focus because much of pandemic response was realized through governor executive orders under a declared state of emergency. Among other things, the NCDHHS secretary serves as the legislatively designated state health advisor “direct[ing] the attention of the State to health matters, which affect the industries, property, health and lives of the people of the State.”^3^ Thus, former NCDHHS Secretary Dr. Mandy Cohen^4^ and the department came into focus for its role in institutionalizing and delivering science advice. Dr. Cohen very publicly embodied the role of state health advisor appearing at most press briefings to discuss scientific aspects of the pandemic and spotlight public health guidance. In December 2020, the state’s largest newspaper circulation, *The News & Observer*, honored Secretary Cohen for her contribution to the state as “the face of the state’s fight against COVID-19” (Keister, 2020).

Via Executive Order 116, Governor Cooper created the Governor’s Novel Coronavirus Task Force on COVID-19 co-chaired by the Director of Emergency Management and the State Health Director. The second meeting of the Task Force on March 12, 2020 was televised and available to the public. The meeting mainly consisted of state agency representatives. Beyond this televised meeting the Task Force fell out of public view. At times, a member of the Task Force would be present at a media briefing alongside the governor or secretary, but the broader activities of the group are not publicly documented. Lack of detail about Task Force advising activities may reflect the limits of this study; it may also reflect a low status of the mechanism itself.

The state does not maintain a publicly accessible central location of the sources of science advice on COVID-19 to North Carolina policymakers. Though it may be that some sources maintain their own public interface, the activities remain difficult to track without a central repository. I identified advisory activities by staying abreast of press releases by the governor’s office, watching media briefings given by the governor and secretary, internet searches, and social media feed. Three primary avenues of science advice surfaced^5^: (1) Advice on Epidemiological Models, (2) Advice on Vaccine Distribution, and (3) State Collected Data. As I identified significant avenues of science advice, I requested interviews with those I considered key players.

I conducted 8 semi-structured interviews by virtual conferencing or telephone and used handwritten notes. All those interviewed served in an advisory capacity and/or were responsible for organizing advice. Four of these interviews occurred during 2020. A reviewer comment led to an additional four interviews in early 2022. I attempt to maintain the confidentiality of interview sources. I also collected data from publicly available recordings of media briefings (https://video.pbsnc.org/show/nc-emergency-management-and-weather).

The main period of study focus is January 2020–February 2021. The study period captures the dynamics of the aftermath of the 2020 United States general election. Throughout this work occasional reference to dates beyond February 2021 are mentioned to document how or when a certain matter ended and what it may reveal about the going-ons during the study period. The boundaries provided by this date range serves to limit study to the advisory mechanisms relevant to the initial year of pandemic response and initial vaccine distribution effort.

An apparent limitation of this study is the small number of interviews conducted. This is a product of both the time constraints public leaders worked under during 2020 and disruptions to my own life presented by the pandemic during 2020 and 2021 (see Myers et al., 2020; Kramer, 2020; and Breuning et al., 2021). Nonetheless, the work captures several key processes and conditions of science advice serving as an important state level case study on COVID-19 pandemic response. Though more interviews would make for a richer telling of what happened it likely would not result in a substantively different account.

Through the interview process it became clear that NCDHHS served as the state’s hub for assimilating and organizing scientific information and channeling information and advice to the governor and/or secretary. So the three mechanisms of advice analyzed are a subset of formal and informal advisory mechanisms that fed into the information gathering activities of NCDHHS. As an actively evolving situation new mechanisms of science advice became apparent as this manuscript was prepared. Two of these deserve noting even though they are not discussed further: the ABC Science Collaborative and the North Carolina Policy Collaboratory.

Closing schools and masking children in school upon reopening was a deeply controversial issue in the United States. In March 2021, Governor Cooper signed The Reopen Our Schools Act of 2021 requiring schools opening under certain conditions to partner and submit data to the research and advising initiative, ABC Science Collaborative. The ABC Collaborative has its roots in the summer of 2020 when local school district leaders,

approached faculty from Duke University and the University of North Carolina at Chapel Hill, seeking to better understand the scientific underpinnings of SARS-CoV-2 mitigation strategies and further guide district-specific policies around reopening. In response to this request, faculty… developed the ABC Science Collaborative (Zimmerman et al., 2021).

The ABC Collaborative received substantial press in June 2021 upon reporting to the General Assembly that existing measures for reopening schools “did an outstanding job” limiting COVID-19 transmission and supported the use of masks in schools (ABC Science Collaborative, 2021). The study was useful for debating with the teacher associations about returning to the classroom (Brown, 2021). There is concern that the group’s study was not forthright about the level of uncertainty that remained around the effectiveness of masking children due to study design (Zweig, 2021).

Several years prior to the pandemic in 2016, the General Assembly created the North Carolina Policy Collaboratory at the University of North Carolina Chapel Hill, “to facilitate[e] the dissemination of the policy and research expertize of The University of North Carolina for practical use by State and local government.”^6^ In May 2020, the General Assembly allocated $29 million in public funding to the Collaboratory to support research and activities addressing the pandemic.^7^ The Collaboratory channeled the pandemic funding into 85 projects across 14 University of North Carolina system schools (North Carolina Policy Collaboratory, 2020, p. 19). NCDHHS maintains a close working relationship with the North Carolina Policy Collaboratory (Advisor B,^ 2022^).

At least two Collaboratory funded projects translated into direct science advice. One project, the North Carolina Central University’s Advanced Center for COVID-19 Related Disparities (ACCORD) was important for developing vaccination incentives and improved understanding of obstacles and motivations for vaccination among historically marginalized communities (NCDHHS, personal communication, November 17, 2021). ACCORD was one of the many sources of advice on vaccine distribution received by the department. The other project is a collaboration among multiple North Carolina universities to track community levels of COVID-19 through wastewater monitoring. The resulting data became available in January 2021 through an NCDHHS online dashboard (https://covid19.ncdhhs.gov/dashboard/wastewater-monitoring). Data may be useful to some local health officials to inform communities about local COVID-19 trends.

## The interface of gubernatorial power during a state of emergency and NCDHHS advising activities

States differ in the powers they bestow on their governors. Typically, governor powers become more extensive under state emergency declarations to efficiently organize statewide response. Such declarations also enable flexibility of public spending as states become eligible for emergency federal resources. However, pandemic emergency declarations left governors wielding far reaching authority over a prolonged period. In North Carolina, the governor’s concentrated power interfaced with the roles and responsibilities of NCDHHS elevating the department’s influence and access to policymaking.

Renegotiating the limits of gubernatorial power under an emergency declaration became a focal point in many states including North Carolina (Gardner, 2021). Several lawsuits against Governor Cooper challenged his emergency powers (Ballotpedia, 2020–2021). Though the governor had lifted all statewide pandemic related restrictions on the general public by the end of July 2021, the powers retained by the governor remained a concern. At least among Republican legislators, the prolonged state of heightened executive power undermined basic principles of democratic governance (Moore, 2021). These legislators argued that emergency power was intended to enable efficient response to more acute events such as, hurricanes. However, the ability to reign in the governor was limited by the governor’s veto power and on November 1, 2021, the governor vetoed the Emergency Powers Accountability Act, which would have placed more specified time limits on a declared states of emergency and the associated increased executive power. Later the same month, much of the language limiting emergency power within the vetoed bill was passed as part of the 2021 Appropriations Act.

The governor appoints the NCDHHS secretary who then appoints the state health director, among others within the department. Though there is no required scientific expertize for the secretary, the state health director must be a state licensed physician. The health director has responsibilities related to overseeing disease surveillance, serving as an arbiter of public health threats, and overseeing the implementation of disease spread control measures. The health director has the legislative authority to hold people in isolation or quarantine for up to 30 days beyond which court consent is needed. This authority includes limiting freedom of movement of those who have not received immunizations against communicable diseases if the health director determines that immunization is necessary to control disease outbreak. However, when the governor declared a state of emergency on March 10, 2020 (Exec. Order 121, 2020), he assumed the power to limit public movement until the state of emergency was formally terminated. Between March 2020 and the end of February 2021, the main period of focus for this study, Governor Cooper signed 148 executive orders related to the pandemic and Secretary Cohen^8^ signed 6 orders and one directive (NC, 2021a).

A state of emergency enables agencies flexibility in their processes and organization to accommodate emergency operations. This flexibility proved important for NCDHHS to handle the sudden and immense flow of information about the pandemic into the department. The department development various in house “teams.” As the pandemic “rapidly expanded or exploded” the collaborative structures within the department shifted; “There have been many iterations within NCDHHS in respect to structure, teams, and pillars of response” (Advisor T, 2022).

Teams within the department represented different response concerns and information gathering activities with individual stakeholders and communities. For instance, there were teams that represented historically marginalized groups, migrant workers, and the homeless and each team interacted regularly with community leaders of those groups to stay in tune with what was going on it those populations (Advisor T, 2022). There were teams that met weekly with businesses and hospitals to review information on supply issues, COVID testing modalities, and position papers (Advisor T, 2022). An epidemiology team would summarize new studies in the literature and provide the department with take away messages (Advisor L, 2021).

NCDHHS made use of many external advisory activities especially as it related to vaccine distribution. The department was one of the first among the states to commission a marketing research consultancy to develop public health messaging. Multiple rounds of marketing research resulted in clear findings around fostering trust helping the department develop their vaccine tag line, “You have a spot. Take your shot” (Advisor L, 2021).

The team approach was integral to streamlining advice developed from the vast amount of information that department personnel gathered. Teams had twice a day “stand-up” meetings with the secretary that were a “key channel for communicating important advice straight to the top” (Advisor L, 2021). About three to four times a week there were additional briefings for the secretary on particular issues.

Fluidity in the way NCDHHS organized information gathering and advice included fluidity in the level of interaction between the governor and department senior leadership—generally, those within the secretary’s office. The secretary briefed the governor multiple times a week and often daily (Advisor L, 2021). There was continuity among the governor and NCDHHS senior leadership,

Typically, the secretary is in the cabinet with the governor. But, during the pandemic [NCDHHS Senior Leadership] met often with him. In the beginning [Senior Leadership] had a meeting with the governor every day. Even now we meet with him twice a week… It’s been a very collaborative effort with the group; we’ve had a multi-disciplinary and team approach (Advisor M, 2022).

There was less interaction between senior leadership and the General Assembly. This was at least in part because of the refusal by many Republican legislators to observe the mask mandate and social distancing practices. Senior leadership was concerned about the risk of a COVID-19 outbreak among department personnel that would hobble the state’s ability to respond. Still, Secretary Cohen was regularly available to the General Assembly via web conferencing and made herself available for further questions. The secretary aimed to keep information shared with legislators the same as that shared with the public and legislators regardless of political affiliation valued her transparency (Advisor B, 2022).

## Three avenues of COVID-19 Science Advice for North Carolina Pandemic Response

### Model overview

In March 2020, Governor Cooper issued several executive orders limiting social gatherings, closing businesses, and requiring the public to “stay at home” (Exec. Order 121, 2020). The orders necessarily created a need to plan for lifting the restrictions. At the time, epidemiological modeling had taken center stage across the world as a main source of predictive information about COVID-19 risk. The models were numerous, provided substantially different perspectives about the impact of the virus, and at times, forecasted devastating loss of life^9^ (e.g., Begley, 2020). There was controversy, misunderstanding, and misleading messaging about the meaning of model output (Eker, 2020; Saltelli et al., 2020).

Governor Cooper and Secretary Cohen sought guidance about the epidemiological models by first looking to modeling activities within North Carolina state universities. However, even within the state, modeling activity was diverse. Dr. Cohen reached out to a former executive in her department familiar with data and analytics and already connected to an informal group of epidemiologists in the private sector investigating COVID-19 impacts in North Carolina (Advisor Y, 2020). The existing connections and policymaker interest led to the creation of an “informal and independent” group of 12 North Carolina epidemiologists, data scientists, and public health experts from public and private institutions, and working under the umbrella of the University of North Carolina Sheps Center for Health Services Research. The group examined several epidemiological models to provide policy recommendations on how to lift the stay at home orders (North Carolina Collaborative Modeling, 2020a). Table 1 provides a list of the 12 collaborators and their affiliations.

**Table 1:** Informal Group collaborators and their affiliations at the time Brief 1 (adapted from North Carolina Collaborative Modeling, 2020a).

Collaborator	Affiliation
Bradley Adams, MS	Managing Actuary, Blue Cross and Blue Shield of North Carolina
Rachael Billock, MSPH, Ph.D Candidate	Department of Epidemiology, Gillings School of Global Public Health, University of North Carolina at Chapel Hill
Alex Breskin, Ph.D	Senior Epidemiologist, NoviSci, Inc.
M. Alan Brookhart, Ph.D	Chief Scientist, NoviSci, Inc.; Professor, Duke University School of Medicine
Hilary Campbell, PharmD, JD	Research Associate, Margolis Center for Health Policy, Duke University
Scott Heiser, MPH	Senior Manager, Health Care and Medical Expense Strategy, Blue Cross and Blue Shield of North Carolina
Mark Holmes, Ph.D	Director, Cecil G. Sheps Center for Health Services Research; Professor, Health Policy & Management, Gillings School of Global Public Health, University of North Carolina at Chapel Hill
Sara Levintow, Ph.D, MSPH	Epidemiologist, NoviSci, Inc.; Department of Epidemiology, Gillings School of Global Public Health, University of North Carolina at Chapel Hill
Pia D. M. MacDonald, Ph.D, MPH, CPH	Senior Director and Senior Epidemiologist, RTI International; Adjunct Associate Professor, Department of Epidemiology, Gillings School of Global Public Health, University of North Carolina at Chapel Hill
Aaron McKethan, Ph.D	CEO, NoviSci, Inc.; Senior Policy Fellow, Margolis Center for Health Policy, Duke University; Adjunct Professor, Duke University School of Medicine
Kimberly Powers, Ph.D	Associate Professor, Department of Epidemiology, Gillings School of Global Public Health, University of North Carolina at Chapel Hill
Sarah Rhea, DVM, MPH, Ph.D	Research Epidemiologist, RTI International

This “Informal Group” considered model output under two basic scenarios: (1) lift all restrictions at once or (2) lift restrictions gradually. The group had the initial goal to “buy hospitals some time” to prepare for an increase in COVID-19 cases in lieu of drastic measures such as, converting stadiums into hospital settings (Advisor Y, 2020). Their analysis indicated that lifting restrictions all at once would create a surge of illness that would outstrip hospital capacity within several weeks.

Secretary Cohen asked for “a landscape view” of modeling activity for COVID-19 in North Carolina but there was no remit (Advisor Y, 2020). The lack of a formal remit was helpful for enabling quick production of perceived policy relevant advice. The group was able to take “a lot of liberties in framing questions that could be informative” and chose to focus on “purposefully broad stroke” scenarios so as to respect the models’ limits in granularity of meaningful output.

The Informal Group briefed the North Carolina General Assembly on their findings on Sunday, April 5th. The next day, the Informal Group made their findings public via Brief #1 (North Carolina Collaborative Modeling, 2020a) and it was referenced by Secretary Cohen during a media briefing (NC, 2020a; PBS, 2020a). Secretary Cohen used the Informal Group’s findings in justification of existing policy response,

this morning a collaborative of North Carolina data experts from the private and public sectors released a North Carolina specific modeling forecast looking at how COVID-19 could affect our state in the coming months. The model reinforced the things we’re already doing, the need for social distancing to slow the spread of COVID-19 and ensure that hospital care is there for the people that need it. The team found that the social distancing policies that we currently have in place in North Carolina will help lower the likelihood that we’ll overload our healthcare system. That’s good news. On the flip side. They found that if we ended those social distancing efforts at the end of April it could lead to a greater than 50% probability that we will outstrip our acute and ICU bed capability possibly as soon as Memorial Day. And they also found that if social distancing was to stop at the end of April roughly 750,000 North Carolinians could be infected by June 1st as opposed to around 250,000 if some form of effective social distancing remains in place (PBS, 2020a).

Brief #2, released about 2 weeks later, largely reiterated the findings of Brief #1 but situated the information in an explicit policy context. The group advocated for a specific approach, sought to depoliticize the option, and acknowledged the inherent social value trade-offs in decision making. The Informal Group retitled the scenario of lifting orders all at once as “Flipping a Switch,” and the scenario of lifting orders gradually as the “Dimmer Switch,”

The question now is, “how do we transition from where we are now into a next phase, and how can this transition be guided by data?” As we describe below, this transition plan must ensure that we remain vigilant to suppress viral transmission and discourage significant spread of COVID-19 in the interest of both public health and the economy (North Carolina Collaborative Modeling, 2020b, p. 2)

The Informal Group advocated for implementation of the Dimmer Switch option, “designed to maintain manageable levels of viral transmission while the state calibrates and implements a staged reopening” (p. 3). They comment further that a Dimmer Switch approach will,

put people back to work at an appropriate pace. It will also facilitate carefully monitoring transmission rates and hospital and healthcare workforce capacity so that reopening adjustments can be made in the interest of public health. In practice, the state likely needs a set of multiple dimmer switches—not a single policy approach for the whole population but varying approaches for different populations or geographies.

Brief #2 also acknowledged the political context of the work. Though hospital capacity was a concern across the nation, situating the analysis within this context may reflect the membership of the group (Table 1). They note the bias, “healthcare capacity is just one factor guiding the state’s reopening” (p. 6).

Despite the adamant position about the Dimmer Switch option, the group indicated that many options existed within the approach. They close by noting the difficulty in the situation,

reopening can be calibrated with technical strategies (such as those we describe here) to avoid exceeding hospital capacity, but in the end, any chosen strategy is a set of complex tradeoffs with profound ethical and social consequences (p. 7).

Interestingly, the statement acknowledges the underlying politics while reducing the problem of pandemic response to one of technical capability by proposing the ability to “calibrate” society.

In an April 15 press release, Governor Cooper announced his adoption of a Dimmer Switch approach to lifting orders and shares responsibility for the decision with experts who provided (the inherently value laden) a “dangerous” outlook on disease spread,

Experts tell us it would be dangerous to lift our restrictions all at once. Rather than an on/off light switch, we are viewing this as a dimmer switch that can be adjusted incrementally (quoted in NC, 2020b).

About a week later, Governor Cooper announced a 3-phase plan for lifting restrictions and that policymakers “will look at a combination of metrics to inform decisions to ease restriction” (NC, 2020c; NC, 2020d). Table 2 shows characteristics of each phase. Though neither the Dimmer Switch or the metrics were codified by executive order, the metrics became a center piece for media briefings held by Cooper and Cohen.

**Table 2:** North Carolina’s three phase plan for easing restrictions as described in the informational slides at the April 23, 2020 media briefing.

Phase	Characteristics
Phase 1	• Stay At Home order remains in place, people can leave home for commercial activity • Those retailers and services will need to implement social distancing, cleaning and other protocols • Gatherings limited to no more than 10 people • Parks can open subject to gathering limits • Face coverings recommended in public • Restrictions remain in place for nursing homes and other congregate living settings • Encourage continued teleworking
Phase 2	• At least 2–3 weeks after Phase 1 • Lift Stay At Home order with strong encouragement for vulnerable populations to continue staying at home • Allow limited opening of restaurants, bars and other businesses that can follow strict safety protocols (reduced capacity) • Allow gathering at houses of worship and entertainment venues at reduced capacity Increase in number of people allowed at gatherings Open public playgrounds Continue rigorous restrictions on nursing homes and congregate living settings
Phase 3	• At least 4–6 weeks after Phase 2 • Lessen restrictions for vulnerable populations with encouragement to continue practicing physical • distancing • Allow increased capacity at restaurants, bars, other businesses, houses of worship and • Entertainment venues • Further increase the number of people allowed at gatherings • Continue rigorous restrictions on nursing homes and congregate care settings

It is clear from the dates of media briefings and Informal Group brief releases that policymakers worked closely with the group. The announcement of a Dimmer Switch approach came around the time of the release of Brief #2 in which the Dimmers Switch approach was given a type of scientific justification. Policymakers also asked the Informal Group to advise on which epidemiological model to contract with. The RTI International model became the model North Carolina contracted with because the state could “turn the crank at a production scale in part because [it] was already used for CDC” (Advisor Y, 2020).

The interaction between policymakers and the Informal Group was, to my knowledge, never made publicly explicit. It is unclear to what extent policymakers interacted directly with the Informal Group as they continued to release briefs throughout 2020. However, in a December 8th media briefing, Cooper references the Informal Group directly when asked a question about their brief released the same day, “We’ve been getting information from this group all along, very early in the pandemic and now lately” (PBS, 2020b). Cooper did not explain how the group’s reports weighed into his decisions.

### State maintained COVID-19 statistical data

The most conspicuous form of science advice in North Carolina pandemic response was state collected statistical data on COVID-19 cases (covid19.ncdhhs.gov/dashboard). In the early stage of the pandemic resources for COVID-19 data collection and access were limited and knowledge of what data was relevant for pandemic response was lacking. The ability to collect the data and make it available was developed once the pandemic was in full swing and with the advice of some members in the Informal Group (Advisor Y, 2020).

The April 23 press release, the governor’s office announced his plan to lift restrictions in three phases “once the data show that key metrics are headed in the right direction” (NC 2020e). The four metrics are:

1. Trajectory in COVID-Like Illness (CLI) Surveillance Over 14 Days
2. Trajectory of Confirmed Cases Over 14 Days
3. Trajectory in Percent of Tests Returning Positive Over 14 Days
4. Trajectory in Hospitalizations Over 14 Day

The metrics provided a broad picture of what was going on in communities and the state (Advisor M, 2022). It also provided the public a finite number of things to become familiar with but there was nothing “magical” about these metrics (Advisor T, 2022). The first metric built on existing surveillance activities throughout the state for diseases such as, flu, and served as a leading indicator. The next two metrics are acknowledged to likely be undercounts. The final metric is the lagging edge and reflects policymaker concerns for hospital capacity. Together, the metrics provide information on surges in disease spread: lead up, peak, and wind down.

**Fig. 2:**
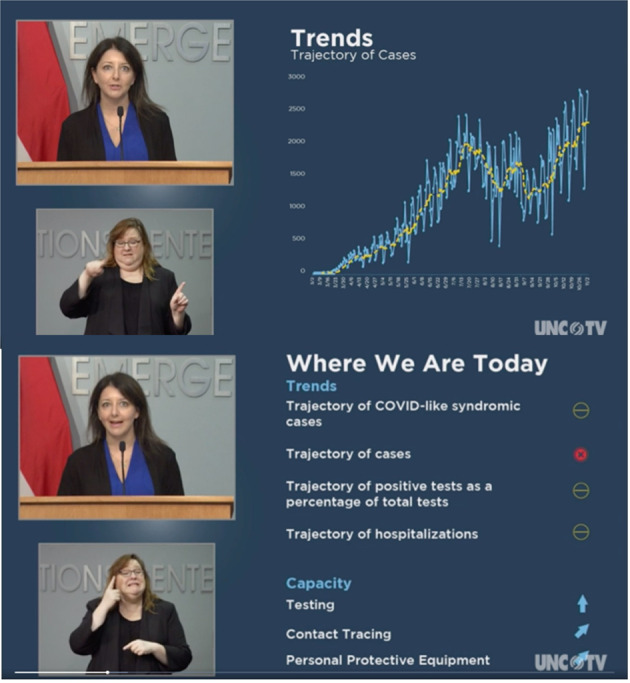
Screen images of the media briefing on November 5, 2020. Secretary Cohen explains COVID-19 data trends during a media briefing. An interpreter translates the message into American Sign Language. This figure is not covered by the Creative Commons Attribution 4.0 International License. Image used under fair use. Fair use allows limited use of copyrighted material without permission for purposes such as scholarship and research. Image courtesy of PBS North Carolina.

Executive orders related to pandemic response often invoked the data and trends in the metrics as grounds for the order but largely in passing. However, the metrics played a very prominent role in the regular media briefings held by one or both, Governor Cooper and Secretary Cohen. Each media briefing began with an account of the numbers of COVID cases, deaths and hospitalizations. Secretary Cohen discussed the data in detail with the aid of presentation slides (Fig. 2). She used a summary slide that describes each of the trends as increasing, decreasing, or stable, and supporting slides that discuss each of these trends in more detail showing the data trends themselves and focusing on different time periods.

Though Governor Cooper and Secretary Cohen invoked the trends regularly and prominently, the relationship between the metrics and decision making was less clear. The lack of clarity became a quibbling matter between Governor Cooper and a reporter during a question and answer session (PBS, 2020c),

Reporter: What is the threshold for moving backwards… Will you give North Carolinians something tangible to understand your decision making…?

Cooper: Well, first all of these metrics are interactive and we want to make sure we are looking at all of them before making decisions on what we need to do next… We hope to be able to see a slowing of the spread and we have not seen a spike of this here like we’ve seen in other states… We are also having more of a focus locally understanding that there are certain areas that are seeing more spread and that is why we’re working with them.

In follow-up, Secretary Cohen echoed the governor,

I think the governor is exactly right. We’re continuing to look at all our metrics. They interact with each other differently and so there haven’t been exact cut points that we have put out in order to make those decisions [about changes in reopening phases].

As the weather began to cool for the fall season North Carolina COVID-19 case numbers increased. Shortly after the election in November, a reporter asked the governor if he was considering moving back from a “pause” in Phase 3 to which he responded, “We certainly don’t want to, but we are going to let the data guide our decisions” (PBS, 2020d). On January 27, 2021 Governor Cooper issued a modified stay at home order that functioned as a curfew (Exec. Order 189, 2021) but the state remained in Phase 3. Governor Cooper lifted the mask mandate and other social distancing measures on May 14, 2021 after the CDC issued new guidance (NC, 2021b).

The regular media briefings were also occasions to center public attention on a political figure running for re-election, an advantage the governor held over his Republican challenger. This provided opportunity for Governor Cooper and Secretary Cohen to express value positions on contemporary political issues and at times, they did so. Often this took the form of directly invoking the symbolic power of science and scientists—a key rhetorical device that divided Republican and Democrats in their public appeals. For instance, on the merits of wearing a mask the governor argued that,

A mask is not political, it’s patriotic. Overwhelmingly scientists say that the way to slow the spread of this virus, one of the best ways is to wear a mask and social distance…. once we get past this election, I think that will help us in our battle to slow the spread of the virus. Because I think we’ll find not using the political excuse but more and more people who would be willing to follow science to do the things that we need to do to slow the spread (PBS, 2020c).

When asked directly how he was going to “depoliticize the pandemic” the governor argued that it would happen on its own, which would enable science to lead decision making,

We won’t have the distraction of this election where the politicizing of the pandemic was central in many ways and wearing a mask, whether you did it or not, seemed to be a political statement. Now we don’t have to worry about that. Hopefully, we can move forward with science and facts and making sure we are protecting the health and safety of North Carolinians (PBS, 2020e).

A notable example of introducing key election issues into pandemic media briefings came from the secretary’s appearance on June 1. Secretary Cohen refers to the Black Lives Matter protests as an inspiration for decisions on pandemic response. She introduced her COVID-19 update as follows,

George Floyd. I can’t say anything else without first saying his name.… I cannot walk in the shoes of any person of color, but I can use my place, privilege, and power to do better. One small down payment on that call to action is how we respond to COVID-19 as a state and as a department (PBS, 2020f).

Six days after the attack on the Capitol building in Washington, DC, Governor Cooper and Secretary Cohen held a media briefing that deviated from those prior by focusing on a message about “truth.” The governor opened the session by linking the threat to expertize and science as a leading cause in the pandemic and the attack,

This assault on our democracy was the result of dangerous rhetoric, lies, and disinformation that spread far and wide. It’s a stark reminder that our words matter… Lies and misinformation have cost lives during this pandemic, as well. Our nation experienced a one-day peak in reported COVID-19 deaths on Thursday with 4,085. More people could be alive today but for dangerous falsehoods that have been spread about the critical importance of masks, social distancing and other common-sense safety rules. Words matter. People listen to leaders and often follow their calls and imitate their actions… Our leaders must listen to science, focus on the facts, and tell the truth with their words (PBS, 2021).

So, while data was invoked in decision making it also provided an ambiguous scientific basis to justify moral decisions about social control measures. Presenting data gave opportunity for policymakers to interact directly and regularly with the public to share public health messages and communicate their position on contentious political issues.

### Ethical guidelines for vaccine distribution

Federal authorities provided greater direction to states on developing vaccine roll-out plans than they had on other aspects of pandemic response. Towards the latter part of 2020, it was evident that a vaccine would soon be available in limited supply. From August thru October, the CDC issued increasingly detailed guidelines directing states to create vaccination plans that prioritized some groups over others. North Carolina had existing ethics guidelines for such an occasion from the 2007 Ethics Report, but NCDHHS considered these unusable^10^ given a recently developed ethics framework by the National Academies of Science, Engineering and Medicine (NASEM, 2020; Advisor X, 2020).

In the first week of September, NCDHHS requested that the North Carolina Institute of Medicine (NCIOM) convene a task force to advise on drafts of the state’s vaccination plan. NCIOM is a quasi-government organization chartered by the General Assembly in 1983 to advise policymakers on medical and health issues. NCIOM was responsible for convening the task force that produced the 2007 Ethics Report. It’s newly created North Carolina COVID-19 Vaccine Advisory Committee (Vaccine Committee) consisted of three co-chairs and about 50 participants representing diverse stakeholder groups such as public health experts, health care providers, advocacy organization leaders, and representatives of essential workers and at-risk populations (NCIOM, 2020a). NCIOM publicly announced the Vaccine Committee on October 5, 2020 and described its purpose to,

provide expert guidance on the development of the plan, support its successful operationalizing, and increase public awareness about vaccination activities, especially for prioritized and historically marginalized populations. Committee members are reviewing the plan with a focus on ensuring a sound base of science, equity, and operational feasibility (NCIOM, 2020b).

Participants spoke a great deal about their work in helping NCDHHS build trust in the plan and the vaccine. As one participant explained,

Anytime you talk about medicine you have to talk about trust… People of color [and historically marginalized groups]—how they have been abused scientifically and medically are important for how they view the medical system. So, there has to be discussions of trust in that regard (Advisor W, 2020).

The core concept of trust ran throughout participants’ understanding of the purpose of the Vaccine Committee’s work. Participants saw the reason for convening a diverse group was so that it could lend guidance “based on our lived experiences” to,

unearth issues not originally considered by policymakers and provide a different set of issues and priorities. For example, the LatinX community can give insight into who is important for overcoming trust issues and why people won’t come forward such as, immigration issues (Advisor X, 2020).

They also understood diversity among the Vaccine Committee members as serving symbolically with the implicit central purpose “to build trust in the [vaccine] recommendations because advise [to NCDHHS] comes from people across the spectrum and lending additional expertize” (Advisor X, 2020). A major topic of focus was building trust in communications, making sure messaging was “culturally and literarily” accurate (Advisor W, 2020)

Underneath all of this [discussion about vaccine distribution plans] is communication because there are issues with vaccine hesitance, trust in the CDC, and people need to make informed decisions (Advisor X, 2020)

Unlike the typical work of the NCIOM, which is to convene advisory committees to provide recommendations to policymakers, the Vaccine Committee provided feedback on NCDHHS draft plans (Advisor X, 2020). The change in usual procedure was in large part due to the quick turn around the state needed. NCDHHS (2020) submitted its final plan to the CDC on October 16, about 7 weeks after contacting NCIOM. The Vaccine Committee process went as follows: (1) NCDHHS presented the Vaccine Committee with a draft plan, (2) the Vaccine Committee considered the plans and submitted questions to the NCDHHS, (3) NCDHHS responded to the questions in their plan revisions. The Vaccine Committee had 24–48 h to respond to each iteration of NCDHHS’ draft plans.

Despite the short turnaround times, and perhaps because of them, the Vaccine Committee worked together without conflict. Advisor X (2020) noted that the NASEM’s high-reputation lent authority to their ethics framework that encouraged compromise among Vaccine Committee participants. Leadership cues from the CDC were considered central to the relatively quick and direct process of developing a vaccination plan,

Development of the vaccine and its distribution is federally led, there is heavy national leadership. On the other hand, there was no national plan for masks or socially distancing. It was almost the total opposite and was deeply politicized (Advisor X, 2020).

The Vaccine Committee had little emphasis on diversity in political ideology and geography. The limits of the committee in this regard were attributed to the short time frame allotted for the whole process. NCIOM recruited to the Vaccine Committee by relying on participants’ professional networks, a type of snowball method.

Unlike NCIOM’s usual advisory practices, the task force meetings were not open to the public nor were meeting notes posted online (Advisor X, 2020).^11^ The NCIOM web page for the task force provides participant information and several press releases. Decisions to keep the meeting closed to the public reflected three concerns. First, the vaccination plan needed a very quick turnaround. Second, the entire process was counter to what NCIOM was accustom; they were used to providing recommendations rather than questions. Third, organizers wanted to manage the “bubble” around the process so that Vaccine Committee participants felt the “freedom to speak” about the highly sensitive topic of prioritizing some social groups over others for life saving vaccines. Ultimately, the Vaccine Committee was “instrumental” in designing the structure, operations, communications, and data needs for vaccine roll-out to ensure equitable distribution (Advisor L, 2021).

## Discussion

### Strengths: mutual respect and data transparency

Two strengths are common in all three advisory processes. First, policymakers and advisors shared mutual respect and trust for each other’s responsibilities and capabilities. Second, transparency in state COVID-19 data diffused much potential conflict about how the virus was spreading throughout the state (Advisor B, 2022). Transparent data and mutual respect limited the potential for political pressure on advisors to produce predetermined outcomes of their deliberations.

Those that I spoke with were mindful of the challenge policymakers faced and the political context in which they worked. They considered it their responsibility to support policymakers in decision making but not to advocate a position. As one Vaccine Committee member explained,

We have to remember our contributions as advisors is advice—exactly that. When this goes to decision makers, they have a lot of other things to consider…We are supportive of decision makers and their efforts to have vaccinations go to those they identify. If I give advice and they didn’t take it, I will still support their plan (Advisor X, 2020).

Advisors also remained mindful about the limits of their expertize, the limits of the state of knowledge, and their role of feeding information into the policymaking process. The Informal Group worked with “humility” and tried to represent themselves as “policy people that know enough about models, their strengths and weaknesses, to make them informative for policy” (Advisor Y, 2020).

This mutual respect was observable between Governor Cooper and Secretary Cohen during media briefings. The former, an attorney, and the latter, a physician, appeared careful about the topics on which each commented. Governor Cooper commented on state policies, executive orders, and negotiations between the state and federal government. Secretary Cohen focused her comments on data interpretation, a clear public health message, and challenges arising around disease spread and impacted populations. It was not that the two never had overlapping commentary. Rather, each clearly respected the other’s area of expertize and its limits.

The Informal Group was thankful that neither legislators nor the governor pushed them to make model output be more detailed than the group’s judgment warranted. Similarly, the Vaccine Committee felt the advisory process went well because policymakers approached advisors with “open ears and open mind” and an earnest interest in equitability (Advisor Z, 2020). As a result, the Vaccine Committee felt respected. Respect is important in these activities not just for fostering trust but also because the advisory work was pro bono. Participants were happy to oblige their policymakers because they felt their work was meaningful.

Data transparency was important in reducing the potential for conflict. Vaccine Committee participants agreed about the high quality of state COVID-19 statistical data and what it indicated about which social groups experienced a higher incidence of exposure to the virus and adverse outcomes of the disease. Said one participant, “people understood the evidence base for decision making, it wasn’t just guided by gut feelings” (Advisor Z, 2020). Mutual respect and data transparency undoubtably lent to limiting public debate to disagreement about policymaker response as opposed to debating the integrity of science and science advising processes in the state.

North Carolina 2020 election results may serve as a testament to the ability of advisors to protect trust in policymaking about pandemic response. Governor Cooper won re-election over the Republican opponent by 4.51 percentage points—a substantial increase over his 2016 election margin when he won by about 0.2 percent. Yet, by only 1.34 percentage points Republican incumbent President Trump won North Carolina against Democrat candidate Joe Biden. The results indicate that many North Carolina voters supported Governor Cooper and President Trump simultaneously. Since party allegiance does not explain the election results, the pandemic was a central issue in the election, and much of pandemic response was handled by governors the results suggest that North Carolina voters were generally satisfied with the way Governor Cooper handled pandemic response in the state.

### Weaknesses: obscure processes

#### Informal Group

The Informal Group presented dynamic positioning in their advice. The group provided a rather straightforward response about the state of epidemiological modeling: collectively, the models indicate that “reopening” all at once will outstrip hospital capacity. In this way the group acted as Science Arbiters. The group did much the same in later reports responding in positivistic terms to their perceived relevant questions about virus spread and hospital capacity. However, the Informal Group also took a very clear Issue Advocate position for a Dimmer Switch approach to social distancing and stay at home orders in Brief #2. It may seem intuitive for the Informal Group to encourage the Dimmer Switch policy given the alternative modeled outcomes of the Flipping the Switch policy. However, this intuition assumes at least three conditions: policymakers equally valued predictive model information, hospital capacity should be the primary concern for policymaking, and implementation of a Dimmer Switch policy was politically feasible.

Republican legislative efforts to overturn executive orders indicates that model information (and later, the four metrics) did not matter to them in the same way that it did to the governor.^12^ The concern for hospital capacity vied with concern for child well-being when early orders to close K-12 schools made it painfully apparent the outsized role schools have in caring for the nation’s children. “Dialing” safety guidelines resulted in social acrimony. By late February 2021, Governor Cooper had yet to move backwards in the state’s phases, but within phases he created pauses, half-phases, modified stay at home orders, and restriction easing. Each of these changes speaks to the political infeasibility of doing anything other than moving forward in phases. Later reports from the Informal Group sat firmly in the realm of arbiter, though it is unclear whether the questions being asked of the model originated from within the group reflecting their own concerns or with policymakers.

#### Data

Without a firm commitment to how the data would lead decision making the connection between policymaking and data as advice was murky. Some of those I spoke with were adamant that metrics were key to decision making about ramping up and easing restrictions, “We use those metrics to guide policy. We really do.” (Advisor M, 2022). However, at the same time as interviewees recognized the complex value trade-offs inherent in pandemic response decision making they would revert back to the data to defend the political decision, “Ninety-eight percent of conflict was over policy. People felt basically, ‘we should look more like Florida.’ But, unfortunately, the data doesn’t support those types of decisions” (Advisor B, 2022). Thus, the data and metrics served to inform policy but also served to reframe moral judgment about what to do as an exercise in data analysis.

Transparent as it was the data served as a type of Rorschach test amendable to most any political narrative particularly because the two dominant opposing groups did not value the data in the same way. When Dr. Cohen reported on the metrics in press briefings she was able to hold the position of Science Arbiter without the information undermining Governor Cooper’s policy decisions. As to how the Secretary’s interpretation of the data was translated to advice for the Governor behind the scenes before these briefings and what role she played (e.g., Honest Broker vs. Issue Advocate) is likely to remain unknown to anyone beyond their closest confidants.

Governor Cooper was by no means the only policymaker to fall into the fallacy of “following the science” or “letting data guide decision making” (e.g., Sasse et al., 2020). Decisions require making choices about value prioritizations. In hyper-politicized contexts opposing groups each have separate information they are leaning on to support their preferred policies (Sarewitz and Rayner, 2021). This dynamic nearly defined the opposing positions of Democrats and Republicans on pandemic response. Democrats sought government controls to limit virus spread; Republicans did not want that type of government intervention. Statistical data and scientific expertize was leveraged to support Democrats’ policy positions; Republicans invoked other value priorities and information to support policy preferences. Indeed, reframing pandemic response as a technical exercise to be led by scientists fit the narrative promoted by national leaders of the Democratic party (Hilgartner et al., 2021).

#### Vaccine Committee

Development of the Vaccine Committee benefited from an institutionalized mechanism, the NCIOM, and a remit. The policymaking goal to operationalize a vaccination plan especially among historically marginalized populations aligned with a rise in public concern about health care inequities made glaringly apparent by the pandemic. The Vaccine Committee interpreted their charge as directing policymakers towards opportunities to build public trust in the vaccine plan. Presumably, the iterative back and forth between policymakers and the committee ensured that the feedback the committee offered was relevant to the decision context. The Vaccine Committee appears to be a good example of an Honest Broker by identifying the available to policymakers to satisfying policy goals of building public trust among historically marginalized people and provide operational efficiency.

However, as North Carolina implemented the vaccination plan it ran into logistical challenges in some counties. A trade-off became apparent between equitable distribution—geographically and among groups—and county distribution capacities. Secretary Cohen commented on this challenge noting, “a tension between speed and equity” (quoted in News and Observer, 2021). As well, K-12 teachers were deprioritized relative to more vulnerable populations, which became problematic when they voiced reluctance to go back into the classroom (Mareno, 2021). However, the issue about teacher prioritization was rather short lived. About a week after news reports of teacher disgruntlement Governor Cooper announced that vaccines would be available for teachers and childcare workers in about 2 weeks—prioritizing them over other “frontline essential workers” (NC, 2021c). Just over a month later the vaccine became available to the general public (NC, 2021d).

## Politics, public health, and pandemic response in North Carolina

Pandemic response required balancing an enduring tension in American politics: limiting government intervention into private lives and mobilizing government to provide for the public welfare. That science advice on pandemic response fed into this fundamentally political space was not lost on the advisors I spoke with:

Public health advising can’t just take into account just the literature—but what is the risks and benefits to society in the context of schools, economy, community… [Decision making] is about science, politics, public health, and pragmatism… It is such a complex advising and decision process, taking place within an evolving landscape (Advisor M, 2022).

Also not lost on advisors was the deep partisan polarization in which they worked. One advisor suggested that it was perhaps the “original sin” (Advisor T, 2022) of the pandemic to have occurred during an election year.

Because of this unavoidable mixture of science and politics, advisors considered it ideal that there was an inclusive and collaborative decision making process among themselves, the secretary, and the governor. Advisors were proud of their work and proud of the governor. They felt that the governor was “very concerned about making the right decision from a public health standpoint” (Advisor M, 2022) and “anguished” (Advisor Y, 2020) over the economic implications of social distancing mandates. Data transparency and equitable vaccine access were leading concern in NCDHHS, and they delivered well on both fronts (Wong et al., 2021).

But the very same close-knit, collaborative advising and decision making process that advisors credit with a science driven, balanced pandemic response also infringes on ideals of transparent and independent science advice (see also Christensen and Lægreid, 2022b). Much of NCDHHS senior leadership includes political appointees and those directly engaged with policy development. Their support for the secretary and the governor must be considered alongside a shared political alignment (Guston and Klein, 2010). Given the concentrated power the governor wielded during the emergency declaration and the close interaction between advisors and policymakers, advising and decision making do not appear as wholly separate processes. What is more, the most high-profile advisory mechanisms the governor created, the Task Force, virtually disappeared from public view shortly after its creation.

Looking beyond the activities of individuals in the pandemic response context, a broader social and political network begins to take shape. After leaving NCDHHS Dr. Cohen took a position with private industry working alongside a former member of the Informal Group (Aledade, 2022). A member of NCDHHS leadership had a former working relationship with the marketing consultants used to advise on pandemic response communications (NC Early Childhood Foundation, 2018). An influential member of the Vaccine Committee works with a think tank, COVID Collaborative (2022), alongside the director of the Duke (University)-Margolis Center for Health Policy. Duke-Margolis played an important role in advocating for North Carolina needs during the pandemic via a relationship with the National Governors Association (Advisor L, 2021). Such relationships reflect professional networks built over time; we work and recommend for positions those we respect and worked with in the past. At the same time, a close community of expertize and power can indicate the emergence of a policy subsystem that supports shared assumptions about how things are and how things should be (Howlett, 2002).

## Conclusions and recommendations

The failed federal leadership in creating a central message on knowledge about the COVID-19 pandemic undermined North Carolina’s ability to respond in the early stages of the pandemic. NCDHHS confronted the knowledge void by mobilizing a diverse array of information gathering and advising processes. Transparency around data was excellent and the department is proud of their delivery of equitable vaccine access. Mutual respect and trust were apparent between advisors and policymakers. As the official state health advisor, Secretary Cohen developed a very public facing role.

The complex advisory processes between NCDHHS and the governor remains opaque. Interview data demonstrate that advising to the governor did not sit with the secretary alone. A birds-eye view of advising reveals a close network of individuals with incredible access to power through the governor acting under an emergency declaration. It is unclear how much direct influence Secretary Cohen exercised in pandemic response decision making though it seems she may have had quite a bit. The credit due to NCDHHS for “good” science advice is a result of the individuals involved. Different people with different ideals could have produced dramatically different advisory mechanisms and potentially, an altogether circumvention of science advice.

How data metrics translated into decisions is difficult to discern. This is at least in part because despite the governor’s commitment to leading with science one cannot derive policy preferences from data alone. Responsibility for moral judgments about social control measures were deflected to technical artifacts—trends in the metrics—without defining how trends would compel decisions. Given the state of partisan polarization in the United States and the impact this has had on how governors act it would be naïve to assume that party loyalties did not influence advising and decision making or, at least, weigh on the minds of those involved.

This observation is not a critique of the integrity of the advice itself or the people involved. Rather, it reflects the consideration that if there was inappropriate political influence on advisory processes it would be difficult to know because much of the activity was obscure. There are several reasons why advising is difficult to track: time constraints, the sheer extent of advising activities, frequent interaction with key policymakers, and highly sensitive issues. These reasons have merit while also highlighting the need to bolster opportunities for public accountability of its science advisors and policymakers.

In the future, the time limiting of governor powers under a state of emergency will create the need to bring advising out of NCDHHS and into the public realm to influence decision making. However, and importantly, without designating a high level and publicly accountable advisory group(s) the mechanisms that pop-up will be vulnerable to politicization and dysfunction. Partisan polarization and the current temperament of federalism means that even if the federal government can muster a unified voice on the state of knowledge about a pandemic, a state’s politics may render its government unreceptive. It is imperative that North Carolina policymakers institutionalize robust, independent, and transparent science advisory mechanisms to inform response to a pandemic emergency. I make five recommendations towards this end.

1. *Create a standing science advisory committee to serve the governor during times of health crises such as a pandemic*.
  NCIOM may be an ideal institution for managing this type of committee. It has a practiced history in advising on public health and it is both separate from the government and accountable to it. Indeed, the NCIOM is currently working with a similar institution in South Carolina to develop pandemic response recommendation (Carolinas Pandemic Preparedness, 2021). The committee may create subcommittees or separate committees with additional members to address specialized knowledge needs and collaborate with NCDHHS. Another option is to establish a committee within NCDHHS with a mixture of members that are civil servants and non-governmental experts. The committee should have clear requirements to provide reports and recommendations that are publicly accessible. However, the standing committee is created it should be recognized as an authoritative source for providing a cohesive message on the state of knowledge about a pandemic and should remain current so as to not develop advisory products that are outdated when needed.
2. *Separate the roles of state health advisor and NCDHHS Secretary*
  The secretary holds powers and influence in respect to public health policymaking, which creates the potential for conflict between science advisory responsibilities and the policy preferences of the governor. Separating the roles of health advisor and secretary limits this potential conflict of interest. One option is to relocate responsibility for selection of the health advisor. For instance, a bipartisan committee in the General Assembly might create a short list of advisor candidates from which the governor may choose.
3. *Utilize high-profile advisory mechanisms when they are created*
  The Governor’s Task Force virtually disappeared from public view shortly after its creation. It would have otherwise been a powerful means of delivering publicly accessible advice to the governor on a range of pertinent issues. In the future, once a task force is created it should be used. If it is later considered to be a poor fit for policymaker needs it should be disbanded and an explanation offered.
4. *Develop a repository of advisory sources*
  It is commendable that NCDHHS mobilized the state’s rich source of diverse expertize to advise on decision making. However, transparency is needed around where information comes from and why. NCDHHS can develop a central repository for documentation on the advice solicited and received. The repository should include conflict of interest disclosures on all advisors.
5. *Develop a shared set of principles for science advice*
  A shared set of principles for science advice across state agencies can help establish the core values advisors should keep in mind when making expert judgment. Principles may include standards of scientific integrity and value prioritizations for making statements about public health risks (e.g., Group of Chief Scientific Advisors, 2019). This may also include practical guidance such as how to enroll advisory group members to ensure plurality in knowledge and perspective.

## Data Availability

Data sharing is not applicable to this article as no datasets were generated or analyzed during the current study.

## Footnotes

1 To reduce the potential for confusion this section uses the term ‘national government’ for what is otherwise referred to in this work to as the federal government.

2 After the attack on the US capitol on January 6, 2021, Twitter closed former President Trump’s personal account (@realdonaldtrump) “due to the risk of further incitement of violence” by the president’s rhetoric (Twitter 2021). I use news coverage to reference his public tweets.

3 North Carolina Stat. §130A.

4 Dr. Cohen announced her resignation from office on November 30, 2021.

5 In mid-November 2020, the state began using a COVID-19 County Alert System that “uses metrics informed by the White House Coronavirus Task Force and North Carolina’s key metrics to categorize counties into three tiers: Yellow—significant community spread, Orange—substantial Community Spread, and Red—critical community spread” (NCDHHS, 2021). I did not analyze the role of this system as a process of science advice and do not discuss it further.

6 North Carolina S.L. 2016-94 §11.8.

7 North Carolina S.L. 2020-4 §3.3.

8 This does not include the NCDHHS guidelines that apply to public schools.

9 In February 2021, in addition to their own ensemble model, the CDC listed 36 independent COVID-19 models. https://www.cdc.gov/coronavirus/2019-ncov/covid-data/forecasting-us.html.

10 NCDHHS may have used the 2007 ethics report to guide decisions on the scarce resource plan earlier in the pandemic when there were concerns about the availability of ventilators and personal protective equipment (Advisor X, 2020).

11 In a separate communication, Advisor X (personal communication, June 18, 2021) noted that while “not open to the public the meetings were not exactly closed” as there were two–three times as many people on the meeting calls than committee members.

12 However, Republican legislators were receptive to the advice of the Informal Group during a private briefing session held for the General Assembly (Advisor Y, 2020).
